# BARRIERS TO ORAL HEALTH IN THE OLDER POPULATION

**DOI:** 10.1093/geroni/igz038.3218

**Published:** 2019-11-08

**Authors:** Kavita Sharma, Lynn Tepper, Carol Kunzel

**Affiliations:** 1 Columbia University, College of Dental Medicine, New York, United States; 2 Columbia University, College of Dental Medicine, NY, United States

## Abstract

A strong challenge is posed for patients and their caretakers by the growing need for promoting oral healthcare for this population, as research substantiates the connection between oral health and systemic health. This study identified the major barriers to providing optimal oral care to the older population. Fifty patients aged 60 and over visiting the Columbia University College of Dental Medicine Clinic were administered a questionnaire which reflected possible barriers to oral health care. Statistical analysis of data revealed that the top three barriers in order of relevance were the (1) cost of treatment, (2) anxiety, and (3) transportation. The youngest old (60-69) indicated that the lack of time and conflict with work schedules were additional barriers, while the older sample (70+) experienced obstacles due to disability and illness. When gender differences were analyzed, transportation was the most significant as a barrier among males, and fear/anxiety was most significant for females. Ethnically, non-Hispanics indicated that (1) shortage of time, (2) anxiety, and (3) lack of social/physical support were significant barriers. Findings indicated that even those with Medicaid insurance coverage believed that the cost of dental treatment and caregiving responsibilitieswere major barriers to seeking care. Conversely, even those without dental insurance indicated that disability and illness were barriers to seeking care. This pilot study highlighted various barriers to oral health care and highlighted the need for intervention to address barriers, such as social services, expanded Medicaid coverage, and transportation assistance, to ultimately improve access to optimal oral health care.

